# Correspondence: Reply to ‘Quantitative evaluation of X-ray dark-field images for microcalcification analysis in mammography'

**DOI:** 10.1038/ncomms10868

**Published:** 2016-04-22

**Authors:** Zhentian Wang, Nik Hauser, Gad Singer, Mafalda Trippel, Rahel A. Kubik-Huch, Christof W. Schneider, Marco Stampanoni

**Affiliations:** 1 Swiss Light Source, Paul Scherrer Institut, 5232 Villigen, Switzerland; 2 Institute for Biomedical Engineering, University and ETH Zürich, 8092 Zürich, Switzerland; 3 Department of Gynecology and Obstetrics, Interdisciplinary Breast Center Baden, Kantonsspital Baden, 5404 Baden, Switzerland; 4 Institute of Pathology, Kantonsspital Baden, 5404 Baden, Switzerland; 5 Department of Radiology, Kantonsspital Baden, 5404 Baden, Switzerland; 6 Department of General Energy Research, Paul Scherrer Institut, 5232 Villigen, Switzerland

Scherer *et al.*^1^ claim that the phantom study in our Article^2^ might not reliably model real microcalcifications in human breast and referred to previous publications^3^,^4^,^5^ to emphasize that the scattering signal depends on particle grain sizes. Specifically, they mention Michel *et al.*'s simulation results^6^, which report a larger scattering signal of calcium hydroxyapatite compared with calcium oxalate dihydrate (more generally calcium oxalate in Michel *et al.*), contradicting our phantom results. First, we want to emphasize that our Article focused on isolated, single microcalcifications instead of clustered microcalcifications as studied in Michel *et al.*^6^, which is a special clinical occurrence. The formation process of microcalcifications in human breast is very complex and not fully understood^7^,^8^. To the best of our knowledge, the internal structure of isolated microcalcifications remains unknown. Previous works^3^,^4^,^5^ investigating scattering signals as recorded by grating interferometers are all based on simplified, diluted sphere particles models. While we agree that such approaches can reflect the scattering signal formation to a certain extent, it has not been demonstrated whether they can reliably deal with both types of microcalcifications, especially considering that the two types have different crystalline structures. We are aware of the difficulties of simulating actual microcalcifications in human breast, as well as the potential differences between the simulants we used and the real microcalcifications. The aim of the phantom experiment was to show that for realistically approximated chemical composition (based on Haka *et al.*^9^) and unknown (but very likely different) scattering behaviour of the simulants, our method could discriminate between them with excellent selectivity. The discrepancy between our phantom results and Michel *et al.*'s numerical simulation might well come from the fact that their work is based on the assumption that the calcium oxalate has the same concentration and grain size as the hydroxyapatite. Although this assumption does not hold in real samples^6^, it indeed demonstrates the influence of the microcalcifications's structure on the scattering signal. We are currently working on the further exploration and exact determination of the internal structure of real microcalcifications to better understand its contribution to the recorded scattering signal.

Scherer *et al.* pointed out our mistake in the data processing of biopsy and mastectomy samples. Despite the principle description and corresponding formula in our Article are correct, the contribution of the underlying breast tissue was inadvertently neglected in the computation. In the following, we explain in other words what has been described in the section ‘Non-invasive microcalcifications classification' in the original Article. We introduce the quantity noted as *r-*ratio and defined in equation (4) as 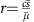, where 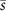 and 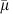 refer to the scattering and absorption signals of the microcalcifications, taking into account the contribution of the underlying breast tissue, while *c* is a system-dependent constant. It follows that 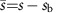 and 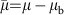, where *s*, *μ*, *s*_b_ and *μ*_b_ correspond to the total scattering, total absorption, breast tissue scattering and breast tissue absorption signals, respectively. The numerical determination of the 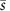 and 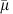 signals is obtained with an image processing algorithm, involving the segmentation of the microcalcification, its localization and the determination of its (projected) size and immediate neighbourhood. When calculating the *r*-ratio, both the absorption and scattering contributions from the underlying breast tissue should be taken into account, but were inadvertently neglected in the original Article. Amended versions of the original Figs 3d and 5f (Figs 1 and 2, respectively) and original Supplementary Fig. 1b (Fig. 3) appear below. The *r*-ratio values for all the microcalcifications shown in Fig. 5f are now found to be distributed in the range of 0.2–9.0 around a peak value of 4.0, while the threshold in the revised original Supplementary Fig. 1 is now set empirically to 11. The broad distribution of the *r*-ratio might suggest a corresponding broad variety of complexity in internal structures within real microcalcifications. The main conclusions of the paper, however, remain unchanged.

Previous works by Bech *et al.* and Schleede *et al.* were cited only indirectly through ref. 25 in the original Article. These two papers should have been cited at the end of the section ‘Non-invasive microcalcifications classification' by adding the sentence ‘The relationship between attenuation and scattering parameters has been discussed in earlier works for material and lung tissue discrimination', where material and lung tissue discrimination refer to refs 10 and 11, respectively.

In summary, we are delighted that our Article has stimulated further investigations of phase-contrast imaging techniques in mammography. We apologize for the oversight in the numerical calculation of the *r*-ratio and for the omission of earlier references. The authors thank and acknowledge Scherer *et al.* for the critical feedback on the results, leading to a correctly scaled *r*-ratio. Although the amendment does not change the overall conclusions of our work, it provides the correct quantitative evaluation of the recorded signals, which will be seminal for upcoming studies by the authors and other groups.

## Additional information

**How to cite this article**: Wang Z. *et al*. Correspondence: Reply to ‘Quantitative evaluation of X-ray dark-field images for microcalcification analysis in mammography'. *Nat. Commun.* 7:10868 doi: 10.1038/ncomms10868 (2016).

## Figures and Tables

**Figure 1:**
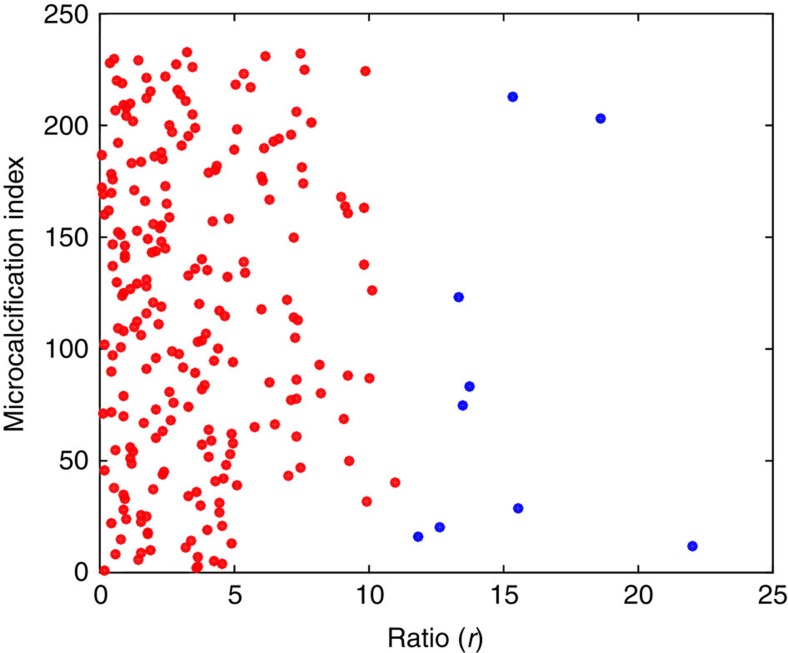
Amended original Fig. 3d. Distribution, with rescaled R-ratio along the ascissa, of all microcalcifications visible in the specimen shown in Fig. 3 of the Original Article.

**Figure 2:**
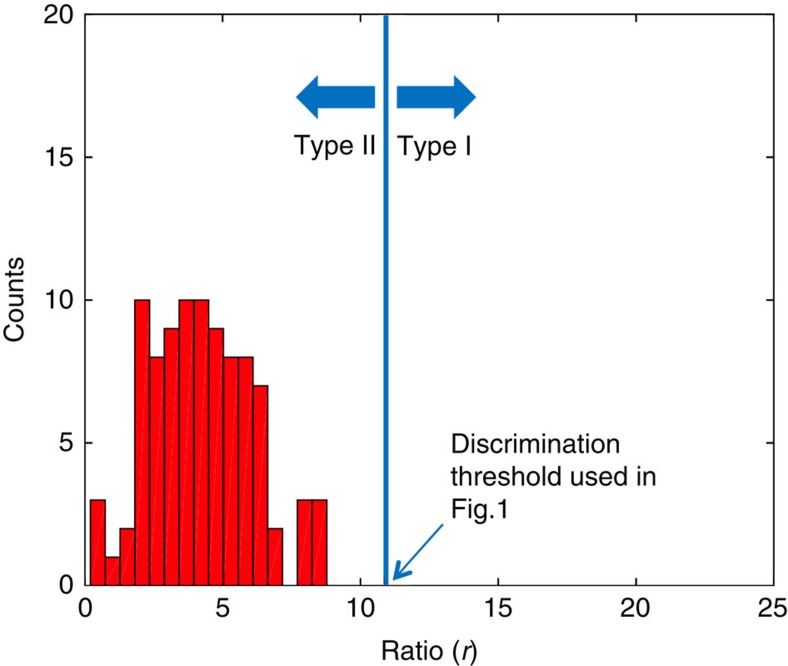
Amended original Fig. 5f. Histogram of the ratio values of the 94 microcalcifications considered on the sample shown in Figure 5 of the original Article. The R-ratio values are distributed in the range of 0.2–9.0 around a peak value of 4.0.

**Figure 3:**
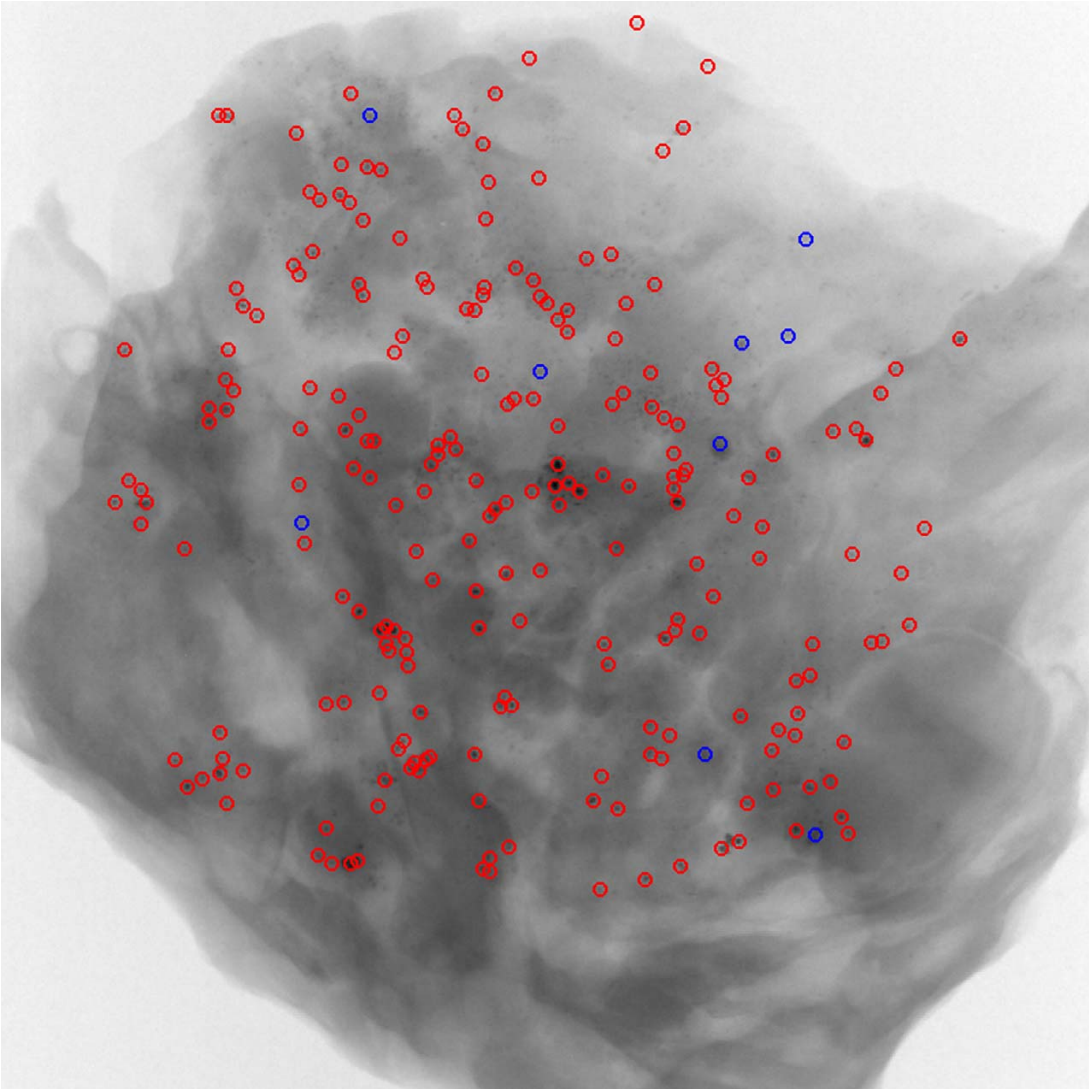
Amended original Supplementary Fig. 1b. Classification of all microcalcifications detected in the sample shown in Figure 3 of the Original Article. The threshold was set empirically to 11, as shown in Figure 1. Accordingly, 9 Type I microcalcifications were found and marked with blue circles while 229 Type II microcalcifications were marked with red circles.
